# Vitamin B12 Deficiency, Hyperhomocysteinemia, and Diabetes as Metabolic Determinants of Cardiovascular Risk in Mexican Women

**DOI:** 10.3390/nu17223535

**Published:** 2025-11-12

**Authors:** Maria D. Ramirez-Villalobos, Eric Monterrubio-Flores, Manlio Marquez-Murillo, Jacqueline Alcalde-Rabanal, Teresa Shamah-Levy, Otilia Perichart-Perera, Nayeli Macias-Morales, Ismael Campos-Nonato

**Affiliations:** 1 Center for Health Systems Research, National Institute of Public Health, Mexico City C.P. 62100, Mexico; dolores.ramirez@insp.mx (M.D.R.-V.); jacqueline.alcalde@insp.mx (J.A.-R.); 2 Center for Nutrition and Health Research, National Institute of Public Health, Mexico City C.P. 62100, Mexico; nmacias@insp.mx; 3 Deputy Directorate of Diagnostic and Treatment Services, National Institute of Cardiology Ignacio Chávez, Mexico City C.P. 14080, Mexico; manlio.marquez@gmail.com; 4 Center for Survey Research, National Institute of Public Health, Mexico City C.P. 62100, Mexico; tshamah@insp.mx; 5 Nutrition and Bioprogramming Coordination, National Institute of Perinatology, Mexico City C.P. 11000, Mexico; oti_perichart@yahoo.com

**Keywords:** cardiovascular risk, vitamin B12 deficiency, hyperhomocysteinemia, type 2 diabetes, scales, national health and nutrition survey, Mexico, risk prediction models, public health, Women’s health

## Abstract

Background: Vitamin B12 deficiency, hyperhomocysteinemia, and diabetes are emerging determinants of cardiovascular risk, particularly among women. Early detection and treatment represent an important public health opportunity to reduce the burden of disease and promote health equity. Objective: We aimed to quantify the prevalence of vitamin B12 deficiency, hyperhomocysteinemia, and diabetes, and to evaluate the potential impact of detecting and addressing these conditions on reducing CVD risk in adult Mexican women. Methods: We analyzed data from 1197 women aged 20–49 years from Mexico’s 2022–2023 National Health and Nutrition Survey (ENSANUT). Serum vitamin B12, folate, and homocysteine were quantified, and 10-year CVD risk was estimated using Framingham and Globorisk models. Population-attributable fractions and cost–benefit analyses were used to assess preventable CVD cases and the economic feasibility of nationwide vitamin B12 supplementation. Results: Nationwide, 37.2% of women have vitamin B12 deficiency, and 30.6% have borderline levels. In Southern Mexico, the prevalence of vitamin B12 deficiency is higher, reaching 52.4%. Elevated homocysteine levels were detected in 12.3% of women. The predicted number of preventable CVD cases ranged from 10,000 to 14,000, and the benefit–cost ratio exceeded 1, supporting economic feasibility. Conclusions: Vitamin B12 deficiency and hyperhomocysteinemia are very common among Mexican women and are associated with an increased cardiovascular risk, especially in those aged 40 to 49. The analysis showed that implementing a national vitamin B12 supplementation strategy could be a cost-effective preventive measure, with a benefit–cost ratio ranging from 1.93 in the base case to 2.98 when broader societal savings are taken into account. These findings highlight the potential of targeted nutritional interventions to reduce the burden of cardiovascular disease in women.

## 1. Introduction

Cardiovascular disease (CVD) remains the leading cause of morbidity and mortality worldwide. Around 17.9 million people died from these conditions in 2019 [1]. By 2030, the number of deaths is projected to rise to 23.3 million. This continuous rise in mortality is expected to place a considerable burden on healthcare expenditures. In Mexico specifically, CVD was the cause of death of 220,000 people in 2021 [2], 44% of whom were women. Ischemic heart disease and acute myocardial infarction are the most lethal CVDs, together accounting for an expenditure of 6.1 billion USD, the equivalent of 4% of the annual healthcare budget for this country [3].

Globally, gender disparities in cardiovascular care remain a major concern. Women are more likely than men to receive an inadequate diagnosis, inappropriate treatment, and delays in receiving appropriate interventions. Although these inequalities have been widely documented in various health systems, their magnitude and determinants in the Mexican context warrant further investigation [4]. These differences may partly explain the disproportionately high burden of cardiovascular morbidity and mortality among women. Furthermore, the risk of CVD in women is markedly higher after menopause, associated with hormonal changes and the high prevalence of obesity, overweight, type 2 diabetes (T2D), hypertension, and dyslipidemia [5].

Factors such as elevated homocysteine (Hcy) levels and vitamin B12 deficiency, conditions that are more commonly seen in adult women, are well-established contributors to cardiovascular risk. However, they remain underrecognized and are often overlooked in clinical practice and in current models of CVD risk prediction [6].

Homocysteine (Hcy), a sulfur-containing amino acid, is known to promote endothelial dysfunction, oxidative stress, and thrombosis, thereby amplifying vascular injury in diabetes [7,8]. Each 5 μmol/L rise in Hcy has been associated with approximately 16% higher CVD mortality [9], and among hypertensive patients, each 1 μmol/L increment has been linked to a ≈7% higher risk [10]. Deficiencies of vitamins B12, B9, and B6 are major contributors to hyperhomocysteinemia [11,12], with B12 deficiency particularly relevant in women and in patients with obesity, T2D or metformin use [13,14].

Deficiency of these vitamins is associated with higher plasma Hcy concentration, as they act as cofactors in its metabolism. B12 participates as a coenzyme in two reactions: the remethylation of Hcy to methionine and the isomerization of L-methylmalonyl-CoA to succinyl-CoA. B12 deficiency is associated with anemia, megaloblastosis, microvascular damage, and neuropsychiatric disorders, which could theoretically be related to the impairment of Hcy remethylation [15]. B12 supplementation has been shown to reduce Hcy levels, with potential benefits in the prevention of cardiovascular events [16].

Cardiac autonomic neuropathy (CAN) is a common complication of diabetes, resulting from oxidative and osmotic stress as well as inflammation affecting autonomic nerve fibers of the heart. CAN has been associated with higher cardiovascular morbidity and mortality, and its effects may be exacerbated by vitamin B12 deficiency, which further impairs nerve function and vascular health [17]. Understanding the interplay between CAN, B12 deficiency, and hyperhomocysteinemia may help identify high-risk women who could benefit from early interventions to reduce cardiovascular events [18].

In Mexico, there is limited evidence on the relationship between serum vitamin B12 levels, homocysteine (Hcy), and cardiovascular disease (CVD). National clinical practice guidelines do not currently recommend B9 or B12 supplementation to reduce CVD risk. Therefore, the objective of this study is to quantify the prevalence of vitamin B12 deficiency, hyperhomocysteinemia, and diabetes, and to evaluate the potential impact of detecting and addressing these conditions on reducing cardiovascular risk in adult Mexican women.

## 2. Materials and Methods

### 2.1. Design and Population

We conducted a cross-sectional analysis using data from the 2022–2023 National Health and Nutrition Survey (ENSANUT), which provides national representation stratified by urban and rural areas and geographic regions [18]. The study population included a subsample of 1197 women aged 20 to 49 years with available blood biomarker measurements, representing an estimated national population of 30.2 million women in this age group. Pregnant or breastfeeding women were excluded due to physiological changes that could influence biomarker levels.

### 2.2. Biomarker Measurements

Venous blood samples (10 mL) were collected from a subsample representing approximately 37% of all women of reproductive age (12–49 years) included in the survey after at least 8 h of fasting. All samples were analyzed at the central laboratory of the National Institute of Medical Sciences Salvador Zubirán (Mexico City, Mexico) [19]. Total plasma homocysteine (Hcy) was measured using a cyclical enzymatic clinical assay with the ARCHITECT system (Architect-i2000; Abbott, TX, USA). Homocysteine levels were categorized as normal (<7 µmol/L), borderline (7–9.9 µmol/L), and high (≥10 µmol/L), based on the consensus of the DACH-LIGA Homocysteine Society [20].

Vitamin B_9_ and B_12_ concentrations were determined using chemiluminescence on a Beckman Coulter Unicel DxI 800 analyzer (Beckman Coulter Inc., Brea, CA, USA). Cutoffs were defined as follows: vitamin B9 deficiency < 4 ng/mL; borderline 4–6 ng/mL [21]; vitamin B12 deficiency < 200.5 pg/mL; borderline 200.5–299.5 pg/mL, based on Brito et al., 2015 [21,22].

### 2.3. CVD and NCDs Self-Report

Self-reported physician-diagnosed cardiovascular disease (CVD) was assessed using ENSANUT questions about myocardial infarction, angina, heart failure, or stroke [19]. Type 2 diabetes (T2D) was identified either by self-report or by laboratory findings, with HbA1c and fasting glucose measurements. Participants were classified as having T2D if they reported a physician diagnosis, or if HbA1c levels were ≥6.5% or fasting glucose levels were ≥126 mg/dL, according to the Mexican Diabetes Guidelines and the American Diabetes Association [23,24].

Hypertension was defined using both self-report and measured blood pressure. Women were considered hypertensive if they reported a prior physician diagnosis or had systolic blood pressure (SBP) ≥ 130 mmHg or diastolic blood pressure (DBP) ≥ 90 mmHg, in line with ENSANUT procedures [19] and European Society of Cardiology recommendations [25].

In addition, trained interviewers collected sociodemographic information to describe the study population and adjust analyses: age, area of residence (urban/rural), a proxy indicator of socioeconomic status, educational attainment, alcohol consumption, and healthcare affiliation [19].

### 2.4. Risk Assessment

All participants with previously diagnosed CVD were excluded from subsequent risk estimations. We then calculated the 10-year risk of developing CVD using the Framingham Risk Score and the Globorisk Risk Scale [26,27]. Both scales incorporate variables such as sex, age, smoking habits, previously diagnosed T2D, use of antihypertensive medication, total cholesterol, HDL cholesterol, and body mass index (BMI), calculated from measured weight and height [19]. Risk estimation was restricted to women aged 40–49 years because both scales are validated only for adults aged 40 years and older, and ENSANUT biomarker determinations required were available only for women aged 20–49 years. The expected number of new CVD cases over 10 years (CVD10y) was estimated by multiplying the average predicted risk for each subgroup by the projected population size for 2029, as reported by the National Population Council (CONAPO) [28].

### 2.5. Estimation of Preventable Cases

**Figure 1:**
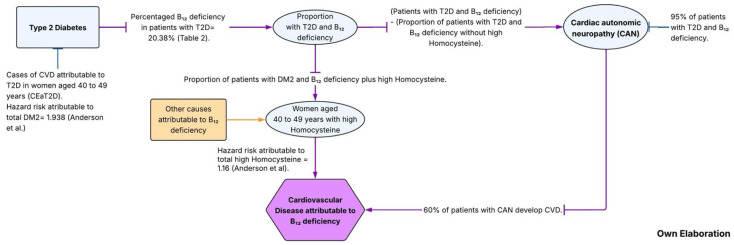
Algorithm of the two metabolic pathways of CVD attributable to B12 deficiency [9].

Finally, we estimated the population attributable fraction (*PAF*%) using the method proposed by Levy *PAF*% [29]. The *PAF*% was then multiplied by the number of cases of CVD10y to estimate the number of preventable CVD cases attributable to high Hcy (HyperHcy) among women aged 40 to 49 years (Figure 1).

Equation used to estimate *PAF*%:

The population attributable fraction (*PAF*%) for elevated homocysteine (HyperHcy) was calculated using the following equation:

$$PAF\%=\frac{Pe\;*\;(RR-1)}{Pe\;*\;\left(RR-1\right)+1}\;*\;100$$

where Pe represents the estimated proportion of women with hyperhomocysteinemia (HyperHcy), and RR = 1.16 corresponds to the hazard ratio reported by Anderson et al. for each 5 µmol/L increase in plasma homocysteine [9]. This continuous effect estimate was applied to approximate the excess risk for categorical high Hcy (≥10 µmol/L), consistent with pooled meta-analytic evidence showing similar relative risks per 5 µmol/L increment (Wang et al.) [30].

Then, to estimate the projected cases of CVD attributable to B12 deficiency among women aged 40 to 49 years with T2D, the following steps were performed:

(1) CVD cases attributable to T2D (CEaT2D):

The population attributable fraction (*PAF*%) was calculated using Levy’s method, where Pe represents the prevalence of T2D and RR = 1.93 (HR), corresponding to the multivariate hazard ratio reported by Anderson et al. [9] for diabetes in patients with coronary artery disease. The resulting *PAF*% was multiplied by the total number of projected CVD10y cases.

(2) Expected cases with T2D and B12 deficiency:

From the total number of CEaT2D cases estimated in step 1, this value was multiplied by the proportion of women with both T2D and vitamin B12 deficiency to estimate the expected number of attributable cases [31,32].

(3) Estimation of Cardiovascular Autonomic Neuropathy (CAN) cases:

We conducted an exploratory scenario analysis for the pathway “T2D + vitamin B12 deficiency → CAN → CVD”. Base-case parameters were set conservatively (CAN progression = 0.60; CAN→CVD = 0.40), with one-way sensitivity analyses over 0.40–0.95 for CAN progression, Ang et al. [31], and 0.30–0.60 for CAN→CVD Pop-Busui et al., [33] (Figure 1).

From the expected number of women with both T2D and vitamin B12 deficiency obtained in step 2, the proportion of cases with hyperhomocysteinemia was subtracted. The remaining value was then multiplied by 0.95, representing the proportion of individuals expected to develop cardiac autonomic neuropathy (CAN) according to published evidence, Ang et al. [31].

(4) Estimation of the number of CVD cases attributable to CAN:

From the number of expected cases with CAN obtained in step 3, the value was multiplied by 0.6, representing the proportion of women with CAN who subsequently develop CVD, as reported in the DCCT/EDIC study Pop-Busui et al. [33] (Figure 1).

### 2.6. Cost-Benefit Analysis

To assess the potential impact of a population-level vitamin B12 supplementation program, we estimated the benefit–cost ratio (B/C) among women aged 40–49 years. The analysis considered two complementary scenarios to capture both direct and indirect benefits to society.

In the first scenario, we estimated the savings to the healthcare system that could result from preventing CVD cases. Based on per capita annual costs of USD 3468 for CVD care [34], we projected the number of cases prevented progressively, assuming a 10% reduction in incidence each year over the intervention period. This scenario reflects the immediate financial benefit to the healthcare system from reducing disease burden.

In the second scenario, the analysis included the healthcare savings estimated in the first scenario, along with additional benefits from reduced informal care needs and productivity losses. It was assumed that 30% of CVD cases are severe; of these, 65% require caregiver support for 8 h per day during the first three months, and 50% continue to require care for up to 12 months. Furthermore, 98% of women aged 40–49 either work outside the home or perform unpaid household labor, meaning that preventing illness also preserves their productive contributions. Caregiver wages and women’s income were calculated using Mexico’s projected 2025 minimum daily wage (278 pesos), converted to USD. Together, this scenario captures the broader indirect benefits of prevention for families and society.

### 2.7. Program Costs

The cost of the vitamin B12 oral supplementation (250 ug per day) program was estimated at 5 pesos (USD 0.25) for a 30-tablet supply (3 USD per person per year). Coverage was modeled among all overweight and obese women in the 40–49 age group (77%), starting with 40% of the target population during the first five years. From the fifth year onwards, coverage progressively increased by 10% annually until reaching 80%. These program costs were then used in combination with the projected benefits (healthcare savings, reduced caregiving needs, and preserved productivity) to calculate the overall benefit–cost ratio.

Calculation of the benefit–cost ratio (VPB/VPC):

The benefit/cost (VPB/VPC) ratio was calculated using the following equation:

$$benefit-cost-=\frac{\sum_{t=0}^{n}\frac{B_{t}}{{\left(1+r\right)}^{t}}}{\sum_{t=0}^{n}\frac{C_{t}}{{\left(1+r\right)}^{t}}}$$

where *B_t_* = benefits per year (VPB), *C_t_* = program costs per year (VPC), *r* = discount rate, *t* = year (from 0 to *n* 10), and *n* = number of years of the intervention (10 years).

### 2.8. Statistical Analysis

Relative frequencies with their corresponding 95% confidence intervals (95% CI) were calculated for key biochemical indicators, self-reported diagnoses, and sociodemographic characteristics. Mean values and 95% CIs were also estimated for biomarker levels, projected CVD risks, and population attributable fractions (*PAF*%). Prevalence estimates with 95% CIs were computed for vitamin B9 and B12 deficiencies, hyperhomocysteinemia (HyperHcy), CVD, and T2D. The prevalence and prevalence ratios of HyperHcy across B_12_ categories were assessed using robust logistic regression models, adjusting for age and B_9_ concentrations. All analyses accounted for the complex survey design and applied appropriate sampling weights. Statistical analyses were performed using STATA version 15 (StataCorp, College Station, TX, USA).

## 3. Results

### 3.1. Characteristics of Survey Population

Table 1 presents the characteristics of women aged 20 to 49 years. The mean age was 35.2 years [95%CI 34.5, 35.9]. Biomarker levels were 7.7 µmol/L for homocysteine (Hcy) [95%CI 7.4, 8.0], 283.9 pmol/L for vitamin B12 [95%CI 263.3, 304.6], and 17.4 ng/mL for vitamin B9 [95%CI 16.7, 18.1].

Table 2 details the prevalence of altered vitamin B9, vitamin B12, and homocysteine (Hcy) levels among women aged 20–49 years included in this nationally representative study. Borderline Hcy was observed in 40.2% [95% CI 35.9, 44.5] of women, while high Hcy affected 12.3% [95% CI 9.2, 15.4]. The highest prevalence of high Hcy occurred among women with hypertension (18.7% [95% CI 9.7, 27.6]). Prevalences between 14% and 15% were observed in indigenous women, those aged 40–49 years, women with primary education or less, residents of rural areas, and women with normal weight. No cases of B9 deficiency were observed. The national prevalence of borderline B9 was 0.7% [95% CI 0.0, 1.5], increasing to 3.7% [95% CI 0.0, 9.7] among women with hypertension.

Borderline vitamin B12 levels were found in 30.6% [95% CI: 26.8–34.4] of women, while vitamin B12 deficiency affected 37.2% [95% CI: 32.7–41.8]. The highest prevalence of B12 deficiency was observed in the southern region (52.4% [95% CI 42.0, 62.9]), followed by women with primary education or less (44.1% [95% CI 32.7, 55.4]), rural areas (42.7% [95% CI 31.2, 54.2]), low socioeconomic status (42.1% [95% CI 33.8, 50.5]), alcohol consumers (38.8% [95% CI 33.4, 44.3]), and women with CVD (36.9% [95% CI 18.9, 54.8]).

### 3.2. Prevalence and Prevalence Ratios

Table 3 shows the prevalence and prevalence ratios of high Hcy according to B12 categories, adjusted for age and B9 concentrations. The prevalence ratio was 2.8 [95% CI 1.2, 6.5] for borderline B12 and 5.2 [95% CI 2.1, 12.8] for B12 deficiency compared with normal B12 levels. This pattern was consistent across all subgroups analyzed.

### 3.3. Increase in CVD Prevalence

We also estimated the increase in CVD prevalence by decades of life using data from 10,774 women, representing 46.3 million women aged 20 years and older nationwide from ENSANUT 2022–2023. A monotonic increase in the prevalence ratio of CVD with age was observed, consistent across all analyzed categories (Table S1).

Nationwide, the average 10-year CVD risk was 3.63% [95% CI 3.0, 4.27] according to the Framingham method, and 2.3% [95% CI 1.8, 2.8] according to the Globorisk method, corresponding to approximately 407,604 and 253,733 projected CVD cases in the next decade, respectively (Table S2).

### 3.4. Estimated Population-Attributable Fractions

Table 4 summarizes the nationwide prevalence of high Hcy (14.8% [95% CI 9.4, 20.3]) and T2D (11.9% [95% CI 9.9, 21.8]) and the estimated population-attributable fractions (*PAF*%) (Table S3). The *PAF*% for high Hcy was 2.3% [95% CI 1.0, 3.9], translating to 9454 preventable cases by Framingham and 5885 by Globorisk. For T2D, the *PAF*% was 12.8% [95% CI 2.0, 23.8], equivalent to 52,282 preventable cases. Considering only women with T2D and B12 deficiency via the cardiovascular autonomic neuropathy (CAN) pathway, an estimated 4411 preventable cases were expected. Overall, the total preventable cases attributable to B12 deficiency, accounting for both high Hcy and T2D via CAN, were 13,865 by Framingham and 8631 by Globorisk (Table S3).

### 3.5. Benefit/Cost Ratio Vitamin B12 Supplementation

Finally, the benefit/cost ratio (B/C) of a population-level vitamin B12 supplementation program for women aged 40–49 years was 1.95 in the first scenario and 2.98 in the second scenario, indicating that the program’s benefits outweigh its costs. The first scenario considered direct healthcare savings from prevented CVD cases, while the second scenario incorporated both these savings and indirect benefits from reduced caregiving needs and preserved productivity, capturing the broader societal impact of prevention (Table S4).

A sensitivity analysis was performed for the first scenario, keeping all parameters constant while varying *t* = 5 years; the B/C ratio was 1.37. When considering *t* = 5 years and *r* = 0.06, the B/C was 1.32. For *t* = 10 years with 60% adherence, the B/C increased to 1.58, while for *t* = 10 years and *r* = 0.6, it was 1.43. When *t* = 10 years with 60% adherence, the B/C was 1.16, and with *t* = 8 years, 60% adherence, and *r* = 0.6, it was 1.04.

In the second scenario, when adherence was set at 60% and other parameters remained constant, the B/C was 2.1. For the same adherence level with *t* = 5 years, the B/C was 1.59. Lastly, with *t* = 10 years and *r* = 0.05 at 100% adherence, the ratio reached 2.8; at *t* = 5 years, the B/C was 2.08.

## 4. Discussion

### 4.1. Summary of the Main Results

This study represents one of the first efforts to quantify the potential impact of vitamin B12 status on CVD risk among Mexican women. Our findings highlight the increased risk associated with hyperhomocysteinemia and B12 deficiency and estimate the projected reduction in CVD cases over the next 10 years among women aged 40 to 49 years, using two validated cardiovascular risk scales: Globorisk and Framingham. Individuals with Hcy elevations attributable to B12 deficiency were excluded from the final risk estimations.

### 4.2. Comparison to Other Studies

The nationwide prevalence of B9 and B12 deficiency observed in our study is consistent with previous reports. Góngora et al., 2023 [35] reported no cases of B9 deficiency and a B12 deficiency prevalence of 34%, while Brito et al., 2015 [21] found a B9 deficiency of less than 5% and a B12 deficiency of 24% among adult women in Latin America and the Caribbean [36]. Comparisons with international data show that average B12 levels in adult women in Austria, Poland, and France were lower than those observed in our study, whereas countries such as Sri Lanka, Italy, and Mexican–American women living in the United States had higher levels [37].

The inverse relationship observed between HyperHcy and B12 is consistent with the existing literature, in which B12 is essential for the conversion of Hcy into methionine, thereby preventing Hcy accumulation in the blood [38].

The high prevalence of B12 deficiency observed in southern regions, among women with low socioeconomic status, lower educational attainment, and in rural populations, can be attributed to low intake of B12-rich foods, reflecting social inequalities as previously described [39].

Our findings regarding the increase in the prevalence ratio HyperHcy associated with B12 deficiency are consistent with evidence reported in the literature [40].

The prevalence of B12 deficiency in women with T2D (20.3%) is similar to that reported by Sauque et al., 2024, in Mexico (19.9%) [41], and by Ouvarovskaiay et al., 2013, in Spain [42]. Higher prevalence of HyperHcy was observed in women with hypertension, which is consistent with previous studies [43]. Elevated blood pressure may result from damage to smooth vascular muscle and endothelial cells, promoting the loss of arterial vasodilation and accelerating the atherosclerotic process [44].

### 4.3. Metabolic Stressors Accelerate Vascular Aging and CVD Events

Multiple pathways link T2D to CVD, including hyperglycemia-related atherosclerosis, inflammation, diabetic cardiomyopathy [45], microvascular endothelial dysfunction, cytokine release, and cellular hypoxia. HyperHcy increases oxidative stress by generating reactive oxygen species and impairing endothelial nitric oxide production, exacerbating vascular dysfunction, particularly in individuals with diabetes or hypertension. These metabolic stressors accelerate vascular aging and CVD events. Vitamin B12 deficiency aggravates this damage by reducing the regenerative capacity of the endothelium, as B12 is essential for DNA synthesis and cellular maturation [44]. Additionally, first-line hypoglycemic agents used for the treatment of T2D may reduce vitamin B12 absorption [45].

Beyond reducing CVD, correcting B12 deficiency may impact the development of cardiac autonomic neuropathy (CAN) in women with T2D [46]. B12 deficiency contributes to microcytic and megaloblastic anemia, intestinal dysbiosis, insulin resistance, hypertension, dyslipidemia, and is associated with cognitive decline and Alzheimer’s disease, given its essential role in DNA synthesis, cellular maturation, and neuronal lipid metabolism [47].

### 4.4. 10-Year CVD Risk Estimates

Differences in 10-year CVD risk estimates between the Framingham and Globorisk scales are expected. The Framingham score may overestimate risk, with variations arising from recalibrations and the absence of Mexican population data [27]. However, the 2011 revision of Mexico’s Clinical Practice Guidelines for the Detection and Stratification of Cardiovascular Risk Factors recommends the use of the Framingham score in the Mexican population, despite its calibration limitations. Globorisk includes Mexican population calibration but tends to underestimate CVD risk, likely due to unrepresentative calibration samples and underestimated dyslipidemia prevalence [48,49]. Both scales may underestimate CVD risk in women, as they do not incorporate reproductive history.

### 4.5. B12 Supplementation Strategy at the Population Level

Based on this evidence, a population-level strategy is proposed for women aged 40 to 49 years, consisting of vitamin B12 supplementation to improve methionine metabolism and reduce Hcy accumulation. Benefit-to-cost estimations indicate that, considering only healthcare system savings, the ratio reaches 1.94 in the base case, with benefits exceeding costs. When including additional societal costs, such as caregiver expenses and lost productivity, the benefit-to-cost ratio increases to 2.98, with overall benefits more than double.

A current challenge in clinical practice is the lack of consensus on optimal cutoff points for defining B12 deficiency and HyperHcy in diverse populations. Establishing population-specific reference values in Mexico could improve early identification and targeted intervention strategies.

Finally, these results support the inclusion of B9 and B12 deficiency and HyperHcy screening in Clinical Practice Guidelines to promote early CVD prevention, reduce female mortality, and lower healthcare costs. Further research is needed to develop more sensitive CVD risk scales incorporating women’s reproductive history as a risk factor.

## 5. Limitations

As a cross-sectional survey, this study cannot establish causal relationships; the findings should, therefore, be interpreted as associations. However, the cardiovascular risk estimations were derived from well-established longitudinal studies, which strengthen the biological plausibility of the observed associations. In addition, the use of ENSANUT data ensured nationwide representativeness of Mexican women. Another limitation is that the study did not evaluate the duration necessary to correct vitamin B12 deficiency or the efficacy of a 30-day supplementation regimen; future studies should evaluate the time and dose necessary to achieve adequate replacement, as well as the safety of supplementation in individuals without deficiency. The estimated progression from type 2 diabetes and vitamin B12 deficiency to cardiac autonomic neuropathy (CAN) and subsequent cardiovascular disease (CVD) was modeled as an exploratory pathway based on clinical studies; therefore, these parameters may overestimate risk in the general population. Finally, risk estimation in this study was restricted to women aged 40–49 years, as the Globorisk and Framingham risk scores are validated for this age range. This age restriction may underestimate the national burden of cardiovascular disease by excluding younger women who may already present early metabolic or reproductive risk factors. Despite these limitations, this study provides robust and nationally representative evidence on the relationship between vitamin B12 deficiency, hyperhomocysteinemia, and cardiovascular disease risk, highlighting critical opportunities for prevention. 

### Strengths

A key strength of this study lies in its use of metabolic quantifications from blood serum, employing robust estimation methodologies. CVD risk estimation was conducted using two internationally recognized methods, which allow for a broader perspective on the estimated impact among Mexican women.

## 6. Conclusions

This study suggests that vitamin B12 deficiency in Mexican women is associated with a higher cardiovascular disease (CVD) risk through two main pathways: hyperhomocysteinemia and autonomic neuropathy. The findings indicate that targeted supplementation in women aged 40 to 49 years—a group at elevated risk—could be a cost-effective preventive strategy, with an estimated benefit–cost ratio of 1.93 for the base case and 2.98 for the alternative scenario. Such an approach may be particularly relevant for women with overweight or obesity, in whom the potential benefits could be amplified.

## Figures and Tables

**Table 1:** Sociodemographic characteristics and mean biomarker values of Mexican women aged 20–49 years by homocysteine levels: ENSANUT 2022–2023 ^a^.

	Total (*n* = 1197)		Homocysteine Normal ^b^ (*n* = 586)		Homocysteine Borderline ^c^ (*n* = 483)		Homocysteine High ^d^ (*n* = 128)	
Characteristics	%	95% CI	%	95% CI	%	95% CI	%	95% CI
All	100		47.4	(43.2, 51.7)	40.2	(35.9, 44.5)	12.3	(9.2, 15.4)
Age group								
20–29	29.6	(25.8, 33.4)	32.2	(26.3, 38.1)	26.4	(21, 31.8)	29.9	(18.2, 41.7)
30–39	34.3	(30.1, 38.4)	32.2	(26.6, 37.8)	38.9	(32.1, 45.7)	27.1	(16.5, 37.7)
40–49	36.0	(31.8, 40.2)	35.5	(29.1, 41.8)	34.6	(28.3, 40.9)	42.8	(32.5, 53.1)
Region								
North	24.7	(19.6, 29.8)	29.3	(22.5, 36.1)	22.6	(16.9, 28.4)	13.7	(6.6, 20.8)
Central	32.3	(27.4, 37.2)	28.8	(23.4, 34.1)	34.8	(28.4, 41.3)	37.8	(24.8, 50.7)
CDMX and surrounding areas	18.1	(14.6, 21.5)	16.9	(12.6, 21.1)	18.1	(13.9, 22.3)	22.8	(13.4, 32.2)
South	24.7	(20.7, 28.7)	24.9	(19.8, 30.0)	24.3	(18.2, 30.3)	25.5	(16.4, 34.5)
Area								
Rural	20.8	(16.4, 25.1)	21.5	(16.4, 26.5)	18.9	(12.9, 24.8)	24.4	(11.1, 37.6)
Urban	28.8	(24.6, 32.9)	27.3	(21.7, 32.9)	29.9	(24.4, 35.4)	30.7	(20.2, 41.1)
Metropolitan	50.3	(45.2, 55.4)	51.1	(44.6, 57.5)	51.1	(44.4, 57.8)	44.8	(33.1, 56.5)
Indigenism								
Yes	3.7	(1.3, 6.1)	2.4	(0.6, 4.2)	4.8	(0.0, 9.8)	5.1	(0.0, 11.8)
No	96.2	(93.8, 98.6)	97.5	(95.7, 99.3)	95.1	(90.1, 100.1)	94.8	(88.1, 101.6)
Tercile of socioeconomic level								
Low	32.3	(27.9, 36.8)	34.7	(28.3, 41.0)	28.9	(22.0, 35.7)	34.5	(21.4, 47.6)
Medium	34.1	(29.6, 38.6)	31.8	(25.9, 37.6)	37.2	(29.7, 44.6)	33.0	(18.4, 47.5)
High	33.4	(28.4, 38.5)	33.4	(26.8, 40.1)	33.8	(26, 41.6)	32.4	(19.8, 45.0)
Educational level (last level completed)								
Primary school or less	16.1	(13.1, 19.2)	13.2	(9.3, 17.1)	18.3	(13, 23.6)	20.2	(10.7, 29.6)
Secondary school	34.4	(30.4, 38.5)	37.2	(30.3, 44.1)	33.5	(26.8, 40.2)	26.7	(16.2, 37.3)
High school or more	49.3	(44.7, 54.0)	49.5	(42.6, 56.3)	48.0	(41.0, 55.0)	53.0	(40.1, 65.8)
Smoking								
No	86.9	(83.8, 90.1)	88.4	(83.6, 93.2)	84.1	(78.9, 89.2)	90.1	(82.7, 97.6)
Yes	13.0	(9.8, 16.1)	11.5	(6.7, 16.3)	15.8	(10.7, 21.0)	9.8	(2.3, 17.2)
Alcohol consumption								
Never	20.0	(16.8, 23.1)	21.0	(15.5, 26.4)	17.5	(12.7, 22.2)	24.6	(14.7, 34.4)
Have not drank in the last year	20.6	(16.9, 24.4)	21.2	(15.1, 27.3)	22.2	(16.7, 27.8)	13.1	(6.9, 19.4)
Alcohol consumption ^e^	59.2	(55.0, 63.5)	57.7	(50.7, 64.7)	60.2	(53.5, 66.8)	62.2	(51.8, 72.5)
Comorbidities								
Hypertension ^f^								
No	87.8	(85.0, 90.6)	87.7	(83.8, 91.6)	89.8	(86.1, 93.5)	82.0	(72.6, 91.4)
Yes	12.1	(9.3, 14.9)	12.2	(8.3, 16.1)	10.1	(6.4, 13.8)	17.9	(8.5, 27.3)
Diabetes ^g^								
No	90.1	(87.4, 92.8)	89.0	(85.2, 92.8)	89.9	(85.3, 94.6)	95.3	(89.0, 101.7)
Yes	9.8	(7.1, 12.5)	10.9	(7.1, 14.7)	10.0	(5.3, 14.6)	4.6	(0.0, 10.9)
Body mass index (BMI) kg/m^2^								
≤25 normal	32.1	(27.0, 37.2)	29.2	(22.0, 36.4)	33.1	(26.6, 39.7)	40.1	(27.6, 52.5)
25–29 overweight	26.6	(22.2, 30.9)	25.5	(19.8, 31.2)	29.5	(22.1, 37.0)	20.9	(11.1, 30.6)
30 ≤ obese	41.2	(36.3, 46.1)	45.1	(38.3, 51.9)	37.2	(30.2, 44.1)	38.9	(24.8, 53.0)
Cardiovascular disease								
No	98.2	(97.5, 98.9)	98.5	(97.6, 99.4)	97.7	(96.5, 98.9)	98.6	(97.3, 100)
Yes	1.7	(1.0, 2.4)	1.4	(0.5, 2.3)	2.2	(1.0, 3.4)	1.3	(0.0, 2.6)
Chronic Renal Failure								
No	99.6	(99.3, 99.9)	99.7	(99.3, 100.1)	99.5	(99, 100)	100.0	(100, 100)
Yes	0.3	(0.0, 0.6)	0.2	(0.0, 0.6)	0.4	(0.0, 0.9)	0.0	---
c-HDL low ^h^								
No	70.2	(66.1, 74.3)	67.7	(61.2, 74.2)	70.0	(64.3, 75.7)	80.3	(71.8, 88.9)
Yes	29.7	(25.6, 33.8)	32.2	(25.7, 38.7)	29.9	(24.2, 35.6)	19.6	(11.0, 28.1)
Institutions of the health system								
Social Security								
No	57.2	(52.2, 62.3)	56.9	(50.5, 63.2)	57.0	(50.2, 63.8)	59.4	(46.6, 72.2)
Yes	42.7	(37.6, 47.7)	43.0	(36.7, 49.4)	42.9	(36.1, 49.7)	40.5	(27.7, 53.3)
Federal Ministry of Health (SS)								
No	43.8	(38.8, 48.9)	43.6	(37.2, 50.0)	45.0	(38.3, 51.7)	40.9	(28.1, 53.8)
Yes	56.1	(51.0, 61.1)	56.3	(49.9, 62.7)	54.9	(48.2, 61.6)	59.0	(46.1, 71.8)
Mexican Institute of Social Security (IMSS) ^i^								
No	62.1	(57.3, 66.9)	61.8	(55.5, 68)	62.9	(56.5, 69.4)	60.7	(47.9, 73.5)
Yes	37.8	(33.0, 42.6)	38.1	(31.9, 44.4)	37.0	(30.5, 43.4)	39.2	(26.4, 52.0)
Institute of Security and Social Services for State Workers (ISSSTE) ^j^								
No	95.2	(93.6, 96.8)	95.3	(93.2, 97.4)	94.0	(91.2, 96.7)	98.7	(96.5, 100.8)
Yes	4.7	(3.1, 6.3)	4.6	(2.5, 6.7)	5.9	(3.2, 8.7)	1.2	(0.0, 3.4)
	Average	(IC95%)	Average	(IC95%)	Average	(IC95%)	Average	(IC95%)
Average age	35.2	(34.5, 35.9)	34.9	(33.8, 35.9)	35.4	(34.3, 36.4)	35.9	(34.0, 37.8)
Biomarkers values								
Homocysteine Umol/L	7.7	(7.4, 8.0)	5.8	(5.7, 6.0)	8.2	(8.1, 8.3)	13.2	(11.7, 14.6)
B12 pg/mL	283.9	(263.3, 304.6)	340.0	(305.1, 374.8)	246.6	(226.1, 267.2)	190.0	(159.1, 220.9)
B9 ng/mL	17.4	(16.7, 18.1)	18.7	(17.7, 19.7)	17.0	(16.1, 17.9)	14.0	(12.4, 15.6)
Triglycerides mg/dL	135.0	(125.8, 144.2)	137.9	(125.8, 149.9)	130.4	(120.3, 140.5)	137.8	(102.5, 173.1)
Cholesterol mg/dL	141.9	(137.4, 146.5)	140.6	(134.8, 146.3)	145.0	(139.7, 150.2)	138.0	(116.0, 160.0)
HDL-c mg/dL	40.8	(39.3, 42.2)	39.5	(37.6, 41.5)	42.2	(40.1, 44.4)	41.0	(37.3, 44.7)
LDL mg/dL	89.4	(86.1, 92.8)	87.9	(83.7, 92.2)	92.3	(87.8, 96.9)	86.3	(75.0, 97.7)
Glucose mg/dL	97.3	(93.4, 101.3)	98.8	(94.6, 103)	97.3	(89.4, 105.2)	91.3	(79.3, 103.4)
HbA1c %	5.5	(5.4, 5.7)	5.6	(5.4, 5.7)	5.5	(5.3, 5.8)	5.4	(5.1, 5.7)
C-reactive Protein mg/dL	0.4	(0.3, 0.6)	0.4	(0.3, 0.5)	0.3	(0.2, 0.5)	0.8	(0.0, 1.9)
Serum Creatinine mg/dL	0.6	(0.5, 0.6)	0.5	(0.5, 0.5)	0.6	(0.6, 0.6)	0.8	(0.5, 1.1)

^a^. ENSANUT 2022–2023: Health and Nutrition Survey in Mexico. All estimates were adjusted for the complex survey design. Confidence intervals (95% CI) may slightly extend beyond 0–100% due to complex survey variance estimation procedures. ^b^. Homocysteine normal: <7 μmol/L. ^c^. Homocysteine borderline: 7 a 9.99 µmol/L. ^d^. Homocysteine high: ≥10.0 µmol/L. ^e^. Alcohol use in the past year or frequently. ^f^. Hypertension: High Blood Pressure (Systolic blood pressure ≥ 130 mmHg and/or a diastolic blood pressure ≥ 85 mmHg and/or with pharmacological treatment for high blood pressure). ^g^. Diabetes: prediabetes (fasting glucose ≥ 100 and <126 mg/dL or HbA1c ≥ 5.7 and <6.5%); diagnosed diabetes (fasting glucose ≥ 126 mg/dL or HbA1c ≥ 6.5% without previous diagnosis). Diabetes: diagnosed + undiagnosed. ^h^. c-HDL bajo: (low high-density lipoprotein cholesterol, <50 mg/dL in men and <40 mg/dL in women). ^i^. Institute of Security and Social Services for State Workers (ISSSTE), ^j^. Mexican Institute of Social Security (IMSS).

**Table 2:** Prevalence of altered levels of vitamin B9, vitamin B12, and homocysteine (Hcy) among women aged 20 to 49 years: Ensanut 2022 and 2023 ^a^.

	Homocysteine Borderline ^b^		Homocysteine Alta ^c^ (*n* = 1197)		B9 Borderline ^d^ (*n* = 1193)		B12 Borderline ^e^ (*n* = 1197)		Deficiency B12 ^f^ (*n* = 1197)	
Characteristics	%	95% CI	%	95%IC	%	95% CI	%	95% CI	%	95% CI
All	40.2	(35.9, 44.5)	12.3	(9.2, 15.4)	0.7	(0.0, 1.5)	30.6	(26.8, 34.4)	37.2	(32.7, 41.8)
Age group										
20–29	35.9	(28.8, 43.0)	12.4	(6.5, 18.3)	2.1	(0.0, 4.5)	32.2	(25.4, 38.9)	42.1	(34.3, 50.0)
30–39	45.6	(38.4, 52.8)	9.7	(5.8, 13.6)	0.2	(0.0, 0.5)	31.7	(24.5, 38.9)	42.0	(34.5, 49.6)
40–49	38.6	(31.7, 45.5)	14.6	(9.3, 19.9)	0.1	(0.0, 0.4)	28.2	(21.7, 34.7)	28.7	(21.8, 35.5)
Region										
North	36.8	(28.8, 44.9)	6.8	(3.5, 10.1)	0.0	(0.0, 0.1)	35.5	(28.9, 42.2)	30.1	(23.1, 37.2)
Central	43.3	(36.3, 50.4)	14.3	(7.7, 21.0)	0.7	(0.0, 1.5)	32.2	(25.2, 39.2)	36.8	(29.0, 44.6)
CDMX and surrounding areas	40.2	(28.1, 52.2)	15.5	(6.4, 24.6)	1.8	(0.0, 5.5)	27.2	(16.6, 37.8)	27.1	(18.1, 36.1)
South	39.5	(31.0, 47.9)	12.6	(8.0, 17.3)	0.6	(0.0, 1.3)	26.0	(19.3, 32.7)	52.4	(42.0, 62.9)
Area										
Rural	36.5	(27.6, 45.4)	14.4	(5.4, 23.4)	0.8	(0.0, 1.9)	29.7	(22.1, 37.3)	42.7	(31.2, 54.2)
Urban	41.8	(34.3, 49.3)	13.1	(7.6, 18.6)	0.6	(0.0, 1.2)	26.6	(19.2, 34.0)	46.4	(37.6, 55.3)
Metropolitan	40.8	(34.4, 47.3)	10.9	(7.2, 14.7)	0.8	(0.0, 2.1)	33.2	(28.0, 38.4)	29.7	(24.4, 35.0)
Indigenism										
Yes	52.0	(20.7, 83.3)	16.8	(0.0, 38.7)	0.0	(0.0, 0.0)	11.1	(1.7, 20.5)	65.2	(39.1, 91.3)
No	39.7	(35.5, 44.0)	12.1	(9.0, 15.2)	0.7	(0.0, 1.5)	31.3	(27.5, 35.2)	36.2	(31.9, 40.4)
Tercile of socioeconomic level										
Low	35.9	(28.1, 43.8)	13.1	(7.5, 18.7)	0.5	(0.0, 1.0)	27.8	(21.4, 34.2)	42.1	(33.8, 50.5)
Medium	43.8	(37.0, 50.6)	11.9	(5.4, 18.3)	0.5	(0.0, 1.2)	33.3	(27.1, 39.6)	37.2	(30.1, 44.2)
High	40.6	(31.8, 49.5)	11.9	(7.3, 16.4)	1.2	(0.0, 3.2)	30.5	(23.0, 37.9)	32.6	(26.2, 39.1)
Educational level (last level completed)										
Primary school or less	45.6	(35.0, 56.2)	15.3	(7.1, 23.6)	0.8	(0.0, 1.7)	23.8	(16.2, 31.5)	44.1	(32.7, 55.4)
Secondary school	39.1	(31.3, 47.0)	9.5	(5.0, 14.0)	0.1	(0.0, 0.3)	31.5	(24.7, 38.4)	35.4	(28.0, 42.8)
High school or more	39.1	(32.5, 45.8)	13.2	(8.9, 17.5)	1.1	(0.0, 2.6)	32.1	(26.6, 37.7)	36.3	(29.9, 42.7)
Smoking										
No	37.9	(33.3, 42.5)	13.0	(9.2, 16.7)	0.9	(0.0, 1.8)	30.7	(26.5, 34.9)	36.9	(31.8, 41.9)
Yes	47.8	(34.2, 61.3)	9.4	(2.4, 16.5)	0.0	(0.0, 0.2)	35.7	(24.7, 46.7)	36.1	(23.3, 48.9)
Alcohol consumption										
Never	35.2	(26.8, 43.6)	14.9	(8.1, 21.8)	0.6	(0.0, 1.4)	29.7	(21.8, 37.6)	36.4	(26.8, 46.1)
Have not drank in the last year	43.4	(32.7, 54.0)	7.7	(3.9, 11.6)	0.0	(0.0, 0.2)	25.4	(17.2, 33.6)	33.7	(25.0, 42.4)
Alcohol consumption ^g^	40.9	(35.0, 46.7)	12.8	(8.5, 17.0	1.0	(0.0, 2.2)	32.5	(27.6, 37.4)	38.8	(33.4, 44.3)
Comorbidities										
Hypertension ^h^										
No	40.2	(35.7, 44.8)	11.7	(8.2, 15.3)	0.4	(0.0, 0.7)	31.5	(27.3, 35.7)	39.0	(34.0, 43.9)
Yes	32.9	(22.9, 42.8)	18.7	(9.7, 27.6)	3.7	(0.0, 9.7)	30.2	(20.2, 40.3)	22.9	(14.4, 31.4)
Diabetes ^i^										
No	38.1	(32.9, 43.3)	12.4	(8.4, 16.3)	0.9	(0.0, 1.9)	31.3	(26.8, 35.9)	39.0	(33.2, 44.7)
Yes	38.8	(24.5, 53.0)	5.5	(0.0, 13.1)	0.0	---	20.6	(11.0, 30.2)	20.3	(10.5, 30.0)
Body mass index (BMI) kg/m^2^										
≤25 normal	41.5	(34.5, 48.6)	15.1	(9.4, 20.8)	0.8	(0.0, 1.6)	29.6	(23.8, 35.4)	38.5	(30.6, 46.4)
25–29 overweight	44.7	(35.2, 54.3)	9.5	(4.7, 14.3)	1.5	(0.0, 4.1)	24.0	(16.8, 31.3)	38.7	(30.0, 47.3)
30 ≤ obese	36.3	(29.7, 43.0)	11.4	(6.2, 16.7)	0.2	(0.0, 0.5)	34.7	(28.7, 40.7)	36.0	(29.3, 42.7)
Cardiovascular disease										
No	40.0	(35.6, 44.4)	12.3	(9.2, 15.5)	0.7	(0.0, 1.5)	30.4	(26.5, 34.2)	37.3	(32.7, 41.8)
Yes	51.7	(33.9, 69.5)	9.2	(0.0, 18.8)	0.0	---	42.3	(23.6, 61.0)	36.9	(18.9, 54.8)
Chronic Renal Failure										
No	39.2	(34.8, 43.5)	12.5	(9.2, 15.9)	0.8	(0.0, 1.6)	31.4	(27.5, 35.3)	36.8	(32.3, 41.4)
Yes	56.9	(14.9, 99.0)	0.0	---	0.0	---	23.1	(0.0, 53.6)	37.1	(0.0, 79.4)
Hypercholesterolemia ^j^										
No	39.1	(34.1, 44.2)	14.3	(10.3, 18.3)	1.1	(0.0, 2.2)	30.1	(25.6, 34.7)	40.2	(34.7, 45.8)
Yes	39.5	(31.6, 47.3)	8.2	(4.2, 12.2)	0.0	(0.0, 0.1)	34.2	(27, 41.3)	28.8	(22.3, 35.3)
Institutions of the health system										
Social Security										
No	40.1	(34.8, 45.5)	12.5	(8.1, 16.9)	1.1	(0.0, 2.4)	28.6	(23.7, 33.6)	41.0	(34.4, 47.6)
Yes	40.5	(34.2, 46.8)	11.4	(7.6, 15.2)	0.1	(0.0, 0.4)	33.3	(26.8, 39.7)	32.0	(25.9, 38.1)
Federal Ministry of Health (SS)										
No	41.4	(35.2, 47.6)	11.2	(7.3, 15.1)	0.5	(0.0, 1.1)	33.1	(26.9, 39.2)	33.0	(27.1, 39.0)
Yes	39.5	(34.0, 44.9)	12.6	(8.2, 17.1)	0.9	(0.0, 2.1)	28.7	(23.7, 33.7)	40.4	(33.9, 46.9)
Mexican Institute of Social Security (IMSS) ^k^										
No	40.8	(35.7, 46)	11.7	(7.7, 15.8)	1.1	(0.0, 2.3)	29.1	(24.3, 33.8)	40.4	(34.3, 46.6)
Yes	39.4	(32.6, 46.2)	12.5	(8.2, 16.7)	0.1	(0.0, 0.2)	33.2	(26.4, 39.9)	31.8	(25.2, 38.3)
Institute of Security and Social Services for State Workers (ISSSTE) ^L^										
No	39.8	(35.4, 44.2)	12.5	(9.2, 15.7)	0.7	(0.0, 1.5)	30.4	(26.5, 34.3)	37.3	(32.7, 42.0)
Yes	50.5	(34.6, 66.4)	3.2	(0.0, 8.8)	0.8	(0.0, 2.5)	34.6	(18.6, 50.7)	33.3	(19.7, 46.9)

^a^. ENSANUT 2022–2023: Health and Nutrition Survey in Mexico. All estimates were adjusted for the complex survey design. Confidence intervals (95% CI) may slightly extend beyond 0–100% due to complex survey variance estimation procedures. ^b^. Homocysteine borderline (Hcy) 7 a 9.99 µmol/L. ^c^. Homocysteine high: ≥10.0 µmol/L. ^d^. Vitamina B_9_ borderline (entre 4 y 6 ng/mL). ^e^. Vitamina B12 borderline (entre 200.5 y 299.5 pg/mL). ^f^. Deficit de Vitamina B12 (<200.5 pg/mL). ^g^. Alcohol use in the past year or frequently. ^h^. Hypertension: High Blood Pressure (Systolic blood pressure ≥ 130 mmHg and/or a diastolic blood pressure ≥ 85 mmHg and/or with pharmacological treatment for high blood pressure). ^i^. Diabetes: prediabetes (fasting glucose ≥ 100 and <126 mg/dL or HbA1c ≥ 5.7 and <6.5%); diagnosed diabetes (fasting glucose ≥ 126 mg/dL or HbA1c ≥ 6.5% without previous diagnosis). Diabetes: diagnosed + undiagnosed. ^j^. c-HDL low: (low high-density lipoprotein cholesterol, <50 mg/dL in men and <40 mg/dL in women). ^k^. Institute of Security and Social Services for State Workers (ISSSTE), ^L^. Mexican Institute of Social Security (IMSS).

**Table 3:** Prevalences and prevalence ratios of homocysteine by B12 levels, adjusted for age and B9: Ensanut 2022 and 2023 ^a^.

	Prevalences						High Homocysteine Prevalence Ratio (≥10.0 µmol/L)				
	B12 Normal ^b^ (*n* = 339)		B12 Borderline ^c^ (*n* = 388)		Deficiency B12 ^d^ (*n* = 435)		B12 Normal ^b^	B12 Borderline ^c^		Deficiency B12 ^d^	
Characteristics	%	95% CI	%	95% CI	%	95% CI	RP	RP	95% CI	RP	95% CI
All	3.9	(0.7, 7.0)	10.9	(6.4, 15.5)	20.6	(14.5, 26.8)	1	2.8	(1.2, 6.5)	5.2	(2.1, 12.8)
Region											
North	1.1	(0.0, 2.5)	3.5	(0.7, 6.3)	16.9	(9.0, 24.7)	1	3.0	(0.0, 13.0)	14.7	(3.8, 56.3)
Central	1.7	(0.0, 4.2)	15.8	(7.6, 24.0)	24.3	(11.4, 37.1)	1	9.0	(2.0, 39.1)	13.8	(2.9, 65.2)
CDMX and surrounding areas	9.9	(1.2, 18.6)	17.9	(0.2, 35.6)	23.9	(0.0, 49.3)	1	1.7	(0.0, 4.5)	2.3	(0.0, 10.5)
South	1.3	(0.0, 3.2)	8.5	(0.1, 16.9)	19.0	(10.4, 27.6)	1	6.2	(1.2, 30.8)	13.9	(3.1, 62.3)
Area											
Rural	1.8	(0.0, 5.4)	13.2	(1.8, 24.5)	21.5	(10.3, 32.7)	1	7.1	(0.0, 57.8)	11.6	(1.4, 93.2)
Urban	0.5	(0.0, 1.4)	10.9	(2.4, 19.4)	23.9	(12.8, 35.1)	1	21.8	(3.0, 153.6)	47.7	(7.1, 316.7)
Metropolitan	5.7	(1.3, 10.2)	10.0	(4.1, 15.9)	19.0	(10.8, 27.2)	1	1.7	(0.0, 3.9)	3.3	(1.3, 8.2)
Tercile of socioeconomic level											
Low	1.3	(0.0, 3.3)	3.3	(0.0, 8.2)	26.8	(13.9, 39.6)	1	2.4	(0.0, 18.8)	19.6	(4.1, 93.7)
Medium	5.4	(0.0, 12.4)	14.8	(6.0, 23.6)	13.7	(5.2, 22.2)	1	2.7	(0.0, 9.6)	2.5	(0.0, 10.3)
High	4.4	(0.0, 8.7)	13.5	(4.9, 22.2)	22.0	(10.8, 33.2)	1	3.0	(1.0, 9.5)	5.0	(1.6, 15.1)
Educational level (last level completed)											
Primary school or less	1.1	(0.0, 2.9)	18.6	(4.5, 32.7)	23.2	(9.6, 36.8)	1	16.0	(3.1, 82.5)	20.0	(3.7, 107.3)
Secondary school	2.1	(0.0, 4.6)	8.8	(2.3, 15.3)	15.8	(7.1, 24.5)	1	4.1	(1.0, 16.3)	7.4	(1.9, 29.0)
High school or more	6.0	(0.4, 11.5)	10.0	(3.7, 16.2)	22.7	(14.1, 31.4)	1	1.6	(0.0, 4.3)	3.7	(1.3, 10.5)
Alcohol consumption											
Never	4.1	(0.0, 10.8)	14.8	(3.3, 26.3)	25.2	(12.6, 37.8)	1	3.6	(0.0, 22.7)	6.1	(1.1, 32.8)
Have not drank in the last year	1.2	(0.0, 2.8)	7.2	(0.5, 14.0)	15.7	(5.9, 25.6)	1	5.8	(1.2, 28.0)	12.6	(2.9, 54.4)
Alcohol consumption ^e^	5.2	(0.0, 10.6)	9.7	(3.7, 15.6)	20.7	(12.3, 29.1)	1	1.8	(0.0, 5.1)	3.9	(1.2, 12.2)
Comorbidities											
Hypertension ^f^											
No	0.8	(0.0, 1.6)	9.7	(4.8, 14.6)	21.5	(14.6, 28.4)	1	11.9	(3.7, 38.5)	26.5	(8.7, 80.9)
Yes	18.3	(5.9, 30.7)	19.2	(5.5, 32.9)	16.8	(1.2, 32.3)	1	1.0	(0.0, 2.9)	0.9	(0.0, 2.9)
Body mass index (BMI) (kg/m^2^)											
≤25 kg/m^2^ (normal)	4.2	(0.0, 9.0)	11.9	(3.5, 20.2)	28.0	(14.5, 41.4)	1	2.7	(0.0, 9.8)	6.5	(1.8, 23.7)
25–29 kg/m^2^ (overweight)	6.8	(0.0, 13.8)	7.0	(0.3, 13.7)	13.6	(5.8, 21.4)	1	1.0	(0.0, 3.8)	1.9	(0.0, 6.3)
30 ≤kg/m^2^ (obese)	0.5	(0.0, 1.3)	11.9	(4.0, 19.7)	20.6	(12.4, 28.8)	1	22.8	(4.0, 128.4)	39.6	(7.5, 207.3)
c-HDL low ^g^											
No	3.7	(0.0, 8.0)	10.1	(5.0, 15.1)	24.9	(17.1, 32.8)	1	2.7	(0.0, 9.6)	6.7	(1.9, 22.8)
Yes	4.9	(0.0, 10.5)	12.1	(1.8, 22.5)	9.2	(3.8, 14.6)	1	2.4	(0.0, 9.7)	1.8	(0.0, 6.4)
Institutions of the health system											
Social Security											
No	3.0	(0.0, 7.2)	12.9	(5.5, 20.2)	18.7	(11.1, 26.4)	1	4.2	(0.0, 19.3)	6.1	(1.3, 27.1)
Yes	4.7	(0.2, 9.2)	8.1	(3.2, 13.1)	24.4	(14.6, 34.1)	1	1.7	(0.0, 4.9)	5.1	(1.7, 15.0)
Federal Ministry of Health (SS)											
No	4.7	(0.2, 9.1)	8.3	(3.4, 13.1)	23.1	(13.3, 32.8)	1	1.7	(0.0, 5.1)	4.9	(1.6, 14.4)
Yes	3.0	(0.0, 7.2)	12.8	(5.3, 20.3)	19.3	(11.6, 27.1)	1	4.1	(0.0, 19)	6.3	(1.4, 27.4)
Mexican Institute of Social Security (IMSS) ^h^											
No	2.7	(0.0, 6.5)	12.7	(5.8, 19.5)	17.7	(10.6, 24.8)	1	4.6	(1.0, 20.7)	6.5	(1.4, 28.7)
Yes	5.5	(0.4, 10.7)	7.8	(2.8, 12.8)	27.2	(16.9, 37.6)	1	1.4	(0.0, 4)	4.9	(1.7, 13.7)

^a^. ENSANUT 2022–2023: Health and Nutrition Survey in Mexico. All estimates were adjusted for the complex survey design. Confidence intervals (95% CI) may slightly extend beyond 0–100% due to complex survey variance estimation procedures. ^b^. Vitamina B_9_ normal > 6 ng/mL). ^c^. Vitamina B12 limitrofe (entre 200.5 y 299.5 pg/mL). ^d^. Deficit de Vitamina B12 (<200.5 pg/mL). ^e^. Alcohol use in the past year or frequently. ^f^. Hypertension: High Blood Pressure (Systolic blood pressure ≥ 130 mmHg and/or a diastolic blood pressure ≥ 85 mmHg and/or with pharmacological treatment for high blood pressure). ^g^. c-HDL: (low high-density lipoprotein cholesterol, <50 mg/dL in men and <40 mg/dL in women). ^h^. Mexican Institute of Social Security (IMSS).

**Table 4:** Preventable cases of cardiovascular disease attributable to B_12_ deficiency via the High Homocysteine and Cardiovascular Autonomic Neuropathy: Ensanut 2022 and 2023 ^a^.

	Number of Cases Attributable to HyperHcy * Due to B12 Deficiency		Number of Cases Attributable to T2D ^b^		Number of Cases Attributable to T2D with B12 Deficiency by CAN **		Total Avoidable Cases of CVD	
Characteristics	Framingham ^c^	Globorisk ^d^	Framingham	Globorisk	Framingham	Globorisk	Framingham	Globorisk
All	9454	5885	52,282	32,546	4411	2746	13,865	8631
All								
Region								
North	1629	758	13,861	6447	1169	544	2798	1302
Central	2921	1310	14,161	6353	1195	536	4116	1846
CDMX and surrounding areas	2594	1504	12,695	7359	1071	621	3665	2125
South	2191	818	11,179	4173	943	352	3134	1170
Area								
Rural	1640	535	11,738	3827	990	323	2630	858
Urban	3604	1720	12,765	6091	1077	514	4681	2234
Metropolitan	4083	2110	28,046	14,493	2366	1223	6449	3333
Tercile socioeconomic level								
Low	3364	1697	11,954	6028	1008	509	4372	2206
Medium	3600	1719	23,492	11,218	1982	946	5582	2665
High	2447	1055	18,929	8156	1597	688	4044	1743
Educational level (last level completed)								
Primary school or less	2992	1046	13,454	4705	1135	397	4127	1443
Secondary school	1220	601	24,429	12,043	2061	1016	3281	1617
High school or more	5193	2701	12,717	6613	1073	558	6266	3259
Alcohol consumption								
Never	2695	913	9266	3139	782	265	3477	1178
Have not drank in the last year	1116	489	19,725	8628	1664	728	2780	1217
Alcohol consumption ^e^	5811	3190	21,183	11,628	1787	981	7598	4171
Comorbidities								
Hypertension ^f^							7277	3820
No	5398	2834	22,269	11,690	1879	986	7127	2691
Yes	3911	1477	38,117	14,396	3216	1214		
Body mass index (BMI) kg/m^2^								
≤25 normal	2380	1555	2123	1387	179	117	2559	1672
25–29 overweight	1787	794	17,780	7898	1500	666	3287	1460
30 ≤ obese	4337	1892	34,871	15,211	2942	1283	7279	3175
c-HDL low ^g^								
No	5194	2676	19,916	10,259	1680	865	6874	3541
Yes	3014	1312	31,036	13,506	2618	1139	5632	2451
Institutions of the health system								
Social Security								
No	4448	2045	28,896	13,282	2438	1120	6886	3165
Yes	5194	2517	23,170	11,229	1955	947	7149	3464
Federal Ministry of Health (SS)								
No	5233	2544	22,807	11,089	1924	935	7157	3479
Yes	4389	2009	29,089	13,317	2454	1123	6843	3132
Mexican Institute of Social Security (IMSS) ^h^								
No	4504	2068	31,756	14,578	2679	1230	7183	3298
Yes	5329	2609	20,341	9956	1716	840	7045	3449

* High Homocysteine (HyperHcy). ** Cardiovascular Autonomic Neuropathy (CAN) ^a^. ENSANUT 2022–2023: Health and Nutrition Survey in Mexico. All estimates were adjusted for the complex survey design. ^b^. T2D: Diabetes, prediabetes (fasting glucose ≥ 100 and <126 mg/dL or HbA1c ≥ 5.7 and <6.5%); diagnosed diabetes (fasting glucose ≥ 126 mg/dL or HbA1c ≥ 6.5% without previous diagnosis). Diabetes: diagnosed + undiagnosed. ^c^. Framingham: Number of cases projected to develop CVD in the next 10 years using Framingham Risk Score. ^d^. Globorisk: Number of cases projected to develop CVD in the next 10 years using Globorisk Risk Score. ^e^. Alcohol use in the past year or frequently. ^f^. Hypertension: High Blood Pressure (Systolic blood pressure ≥ 130 mmHg and/or a diastolic blood pressure ≥ 85 mmHg and/or with pharmacological treatment for high blood pressure). ^g^. c-HDL low: (low high-density lipoprotein cholesterol, <50 mg/dL in men and <40 mg/dL in women). ^h^. Mexican Institute of Social Security (IMSS).

## Data Availability

The original contributions presented in the study are included in the article at https://ensanut.insp.mx/encuestas/ensanutcontinua2023/descargas.php (accessed on 15 March 2025). For more information, please contact the corresponding author.

## Supplementary Materials

The following supporting information can be downloaded at: https://www.mdpi.com/article/10.3390/nu17223535/s1.

## Abbreviations

The following abbreviations are used in this manuscript:

B12	Vitamin B12
B9	Vitamin B9
B/C	Benefit–cost ratio
CAN	Autonomic neuropathy
CVD	Cardiovascular disease
ENSANUT	National Health and Nutrition Surveys
Hcy	Homocysteine
HyperHcy	High Homocysteine
HBP	High blood pressure
HR	Hazard ratio
NCDs	Non-communicable diseases
OB	Obesity
*PAF*%	Population attributable fraction
SBP	Systolic blood pressure
DBP	Diastolic blood pressure
T2D	Type 2 diabetes.
